# Analysis and Construction of a Competitive Endogenous RNA Regulatory Network of Baicalin-Induced Apoptosis in Human Osteosarcoma Cells

**DOI:** 10.1155/2021/9984112

**Published:** 2021-07-19

**Authors:** Haifeng Lan, Haiyan Wang, Mi Gao, Guan Luo, Jiahuan Zhang, Erkang Yi, Chunxiao Liang, Xiaoxiao Xiong, Xing Chen, Qinghua Wu, Ruikun Chen, Biting Lin, Dongyang Qian, Wei Hong

**Affiliations:** ^1^Department of Orthopaedic Surgery, The Third Affiliated Hospital of Guangzhou Medical University, Guangzhou, Guangdong, China; ^2^Guangzhou Key Laboratory of Basic and Applied Research in Oral Regenerative Medicine, Affiliated Stomatology Hospital of Guangzhou Medical University, Guangzhou, Guangdong, China; ^3^GMU-GIBH Joint School of Life Sciences, Guangzhou Medical University, Guangzhou, Guangdong, China; ^4^State Key Laboratory of Respiratory Disease, National Clinical Research Center for Respiratory Disease, Guangzhou Institute of Respiratory Health, The First Affiliated Hospital of Guangzhou Medical University, Guangzhou, Guangdong, China; ^5^The Third Clinical School of Guangzhou Medical University, Guangzhou, Guangdong, China; ^6^Department of Orthopaedics, The First Affiliated Hospital, Guangzhou Medical University/Guangdong Key Laboratory of Orthopaedic Technology and Implant Materials, Guangzhou, Guangdong, China

## Abstract

**Background:**

Baicalin is an extract from the traditional Chinese herb *Scutellaria baicalensis* and has the potential to treat osteosarcoma (OS). However, the transcriptome-level mechanism of baicalin-mediated antitumor effects in OS has not yet been investigated. The aim of this study was to analyze the competitive endogenous RNA (ceRNA) regulatory network involved in baicalin-induced apoptosis of OS cells.

**Methods:**

In this study, CCK-8 and flow cytometry assays were used to detect the antitumor effects of baicalin on human OS MG63 cells. Furthermore, transcriptome sequencing was employed to establish the long noncoding RNA (lncRNA), microRNA (miRNA), and mRNA profiles.

**Results:**

Baicalin inhibited MG63 cell proliferation and induced apoptosis. Totals of 58 lncRNAs, 31 miRNAs, and 2136 mRNAs in the baicalin-treated MG63 cells were identified as differentially expressed RNAs compared to those in control cells. Of these, 2 lncRNAs, 3 miRNAs, and 18 mRNAs were included in the ceRNA regulatory network. The differentially expressed RNAs were confirmed by quantitative real-time PCR (qRT-PCR).

**Conclusions:**

By identifying the ceRNA network, our results provide new information about the possible molecular basis of baicalin, which has potential applications in OS treatment.

## 1. Introduction

Osteosarcoma (OS) is the most common primary bone tumor with high mortality and poor prognosis [1, 2]. Although neoadjuvant and postsurgery chemotherapy has improved in recent years, little has changed for patients with metastatic disease, and their long-term survival rate ranges from 25% to 30% [3]. Therefore, the exploration of new diagnostic and treatment strategies to reduce recurrence and improve the survival rate is a major concern.

Recent studies have shown the potential of natural compounds to serve as successful anticancer agents [4–6]. Baicalin, which is an important flavonoid, is found in the roots of the Chinese herb Huang-qin (*Scutellaria baicalensis* Georgi) [7, 8]. Baicalin exhibits a wide range of pharmacological properties, including antioxidative, anti-inflammatory, antimicrobial, and antiproliferative activities [9–12].

Of all these effects, the antimicrobial effect has been recently highlighted, which includes antifungal, antiviral, and antibacterial activities [13]. Baicalin has an important antibacterial effect on food spoilage bacteria. Antimicrobial resistance, infections caused by microorganisms, and food borne diseases mediated by bacteria are all important concerns for human beings. Common pathogens or relevant bacteria include *Escherichia coli* [14–16], *Salmonella* [17], *Staphylococcus aureus* [18–21], coagulase-negative *Staphylococci* [22], *Pseudomonas aeruginosa* [23–25], *K. pneumoniae* [26, 27], *Listeria monocytogenes* [28], and *Vibrio parahaemolyticus* [29–31]. However, the colonization, growth, persistence, and lifecycle of microorganisms should not be limited within a single species or planktonic state. For example, antimicrobial resistance in such pathogens, biofilm formation, and polymicrobial interactions further complicates food safety problems.

In recent years, studies on antimicrobial resistance have highlighted the hazards of the wide use of antibiotics. For example, the wide distribution of integrons, including class 1 integrons in gram-positive bacteria, such as methicillin-resistant *Staphylococcus aureus* (MRSA) [32–34], methicillin-resistant coagulase negative Staphylococci (MRCNS) [35, 36], and *Enterococcus*, has been reported. In addition to class 1 integrons, class 2 integrons were first observed in Enterococcus [37]. In addition to gram-positive bacteria, integrons are even worse in gram-negative microorganisms. Class 1 and 2 integrons are very common in E. coli; however, the occurrence of class 2 integrons has also been reported in P. aeruginosa. Aside from integrons, some other mobile elements have been reported to play important roles in the spread of antimicrobial resistance, for example, SCCmec is the key reason for the emergence of MRSA [38, 39]. Basically, mobile elements make antimicrobial resistance the leading issue for human beings.

Therefore, recent studies on baicalin, which proved its high antimicrobial activity, are of sufficient importance. In addition, baicalin has been proven to be able to inhibit biofilm formation, attenuate the quorum sensing, and reduce virulence. Although some other natural products have also been reported to exert such anti-biofilm effects [30], the antibiofilm effect exerted via interference in QS looks much better. Therefore, baicalin is a natural product with important effects and activities that may aid in the development of antimicrobials but is different from currently existing antibiotics. In addition to its antimicrobial activities, some evidence shows that baicalin has an antitumor effect on OS cells [40–42]. It also has beneficial effects when used in the treatment of several cancers. However, the molecular mechanisms underlying the contribution of baicalin to OS treatment remain elusive.

In the last decade, advances in the genome-wide analysis of the gene expression have revealed that far more of the genome is transcribed than previously anticipated, and the majority of the genome are transcribed into noncoding RNAs (ncRNAs) [43, 44]. Although most studies on ncRNAs are focused on long noncoding RNAs (lncRNAs, with a length > 200 nucleotides) and microRNAs (miRNAs, with <200 nucleotides), they have only recently attracted attention. Studies have shown that hundreds of lncRNAs have been discovered, and some lncRNAs have been correlated with tumorigenesis and malignancy transformation in various types of cancers [45–49]. However, their potential pathogenesis has not been systematically investigated. In 2011, Salmena et al. [50] proposed the competing endogenous RNA (ceRNA) hypothesis, which states that mRNAs transcribed pseudogenes, and lncRNAs could act as natural miRNA “sponges” and inhibit miRNA function by competing with the binding of one or more microRNA response elements (MREs) in complex and comprehensive regulatory networks, leading to pathogenic conditions. The involvement of the ceRNA regulatory network in tumor initiation and progression has been validated in previous studies. However, the specific ceRNA regulatory network in baicalin-treated human OS cells remains to be elucidated.

In this study, the transcriptome sequencing technique was used to profile the response of MG63 cells to baicalin treatment. The regulatory ceRNA networks of lncRNA-miRNA-mRNA were constructed based on the sequencing data and bioinformatic analysis. Our findings indicated that indepth RNA sequencing analysis of ceRNA could be a promising approach for researching the anticancer mechanisms of therapeutic agents.

## 2. Materials and Methods

### 2.1. Cell Culture

The human OS cell line MG63 was purchased from the American Type Culture Collection (ATCC, VA, USA; CRL-1427TM) and cultured in the recommended medium supplemented with 10% fetal bovine serum (FBS; Gibco, USA) at 37°C in a humidified atmosphere with 5% CO_2_. Baicalin (purity, ≥95%, Sigma-Aldrich, St Louis, MO, USA) was dissolved in dimethyl sulfoxide (DMSO) in a 0.4 mg/ml stock solution and diluted to different concentrations with culture medium immediately before use. The same volume of DMSO with a final concentration of 0.1% was used as a negative control.

### 2.2. Cell Viability Assay

Cells were seeded into a 96-well culture plate at a concentration of 5000 cells/well and treated with baicalin (0, 12.5, 25, 50, 100, and 200 *μ*g/ml) for 24, 48, and 72 h. The cells were washed twice with PBS and incubated with 110 *μ*l fresh medium containing 10 *μ*l CCK-8 solution for an additional 3 h. The plates were analyzed with a microplate spectrophotometer at a wavelength of 450 nm (OD450). We calculated the viability with the following formula: cell viability = (OD450 of treated groups/OD450 of control group) × 100%.

### 2.3. Flow Cytometry

The cells were seeded into a 6-well plate at a density of 3 × 10^5^ cells/well and then exposed to baicalin (0, 12.5, 25, 50, 100, and 200 *μ*g/ml). For apoptosis analysis, the cells were harvested and suspended in 500 *μ*l of binding buffer containing 5 *μ*l of Annexin V-FITC and 5 *μ*l of PI, and then the apoptotic cells were analyzed using a FACSCalibur flow cytometer (BD Biosciences).

### 2.4. LncRNA Isolation and High-Throughput Sequencing

To perform lncRNA sequencing analysis, total RNA was extracted using the miRNeasy Mini Kit (Qiagen). The quality and purity of the extracted RNAs were measured using an Agilent 2100 Bioanalyzer (Agilent Technologies). The lncRNA sequencing library was generated using the Ovation Human FFPE RNA-Seq Library System (NUGEN, 0340-32), and the sample input was 20 ng of each total RNA. Finally, we profiled the expression of the lncRNA libraries using HiSeq 2500 (Illumina, Inc., San Diego, CA, USA).

### 2.5. miRNA Isolation and High-Throughput Sequencing

To perform miRNA sequencing analysis, total RNA was extracted using the miRNeasy Mini Kit (Qiagen). The quality and purity of the extracted RNAs were measured using an Agilent 2100 Bioanalyzer (Agilent Technologies). The miRNA sequencing library was generated using the NEXTflex® Small RNA-Seq Kit v3 (Bio Scientific Corporation, NOVA-5132-05), and the sample input was 20 ng of each total RNA. Finally, we profiled the expression of the miRNA libraries using HiSeq 2500 (Illumina, Inc., San Diego, CA, USA).

### 2.6. Analysis of the Differentially Expressed lncRNAs (DElncRNAs)

Raw reads were treated with a custom Perl script to remove the adapters for read quality control. Then, the read quality was inspected using FastQC and SOAPnuke software, and statistical analyses were run. Paired-end reads were aligned to the human genome version GRCh37 using HISAT2 software. StringTie software was used to assemble the transcripts, and the Perl script was used to screen the known lncRNAs. Then, we obtained the resulting known lncRNAs and used CPC and PFAM software to predict novel lncRNAs. Quantitative analysis of lncRNAs and mRNAs was performed using the *R* package Ballgown, and the lncRNA target mRNAs were predicted. After obtaining the differential expression of the targeted mRNA reads, we predicted the targets and performed enrichment analysis. GO (http://www.geneontology. org) and KEGG (http://www.genome.jp/kegg) analyses were performed for the differentially expressed miRNA- (DEmiRNA-) associated genes. A *P* value <0.05 was considered significant.

### 2.7. Analysis of the DEmiRNAs

**Figure 1:**
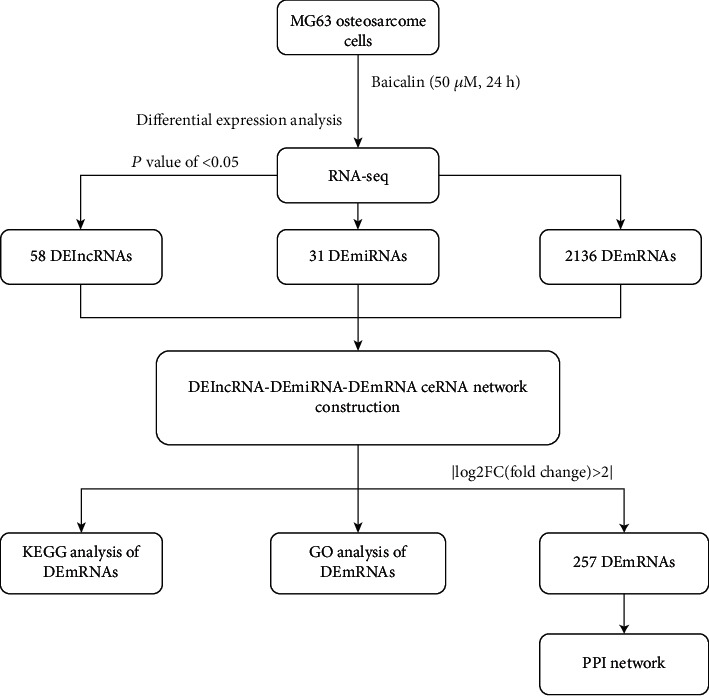
Flow chart of the analytical procedure used in this study.

Raw reads were treated with a custom Perl script to remove the adapters for read quality control. Then, the read quality was inspected using FastQC software, and statistical analyses were performed. Paired-end reads were aligned to the human genome version GRCh37 using Bowtie. We used the reads that mapped to the genome to perform more alignments with different software, including NCGB, Rfam, and hairpin to classify the types of reads. Then, we obtained the resultant known miRNAs and used the unknown reads to predict novel miRNAs. All known miRNAs and novel miRNAs were used to calculate the expression by performing an analysis of variance (AOV) on the transcript per million (TPM) count. After obtaining the differential expression of miRNA reads, we predicted the targets and performed enrichment analysis. GO (http://www.geneontology.org) and KEGG (http://www.genome.jp/kegg) analyses were performed for the DEmiRNA-associated genes. A *P* value <0.05 was considered significant. The top 20 most significant pathways of the upregulated miRNAs and downregulated miRNAs were chosen to construct the pathway relation network, which was based on the interaction data in KEGG. The pathway relation network was used to identify the regulatory effects of these pathways. A flow chart of the analysis procedure is illustrated in Figure 1.

### 2.8. Protein-Protein Interaction (PPI) Network

To understand the interactions of the differentially expressed mRNAs (DEmRNAs), we constructed a PPI network using the Search Tool for the Retrieval of Interacting Genes (STRING, http://string.embl.de/). Combined scores greater than 700 were considered statistically significant. The PPI network was visualized using Cytoscape v 3.6.1 software. Subsequently, hub gene networks were identified by MCODE v1.5.1. In addition, GO enrichment analysis was performed using STRING to functionally annotate the DEmRNAs in the hub gene network.

### 2.9. Construction of the ceRNA Network

A ceRNA network was constructed to discover the ceRNA mechanism based on the differentially expressed RNAs and was established using Cytoscape v 3.6.1 software. First, the lncRNA-miRNA and miRNA-mRNA target relationships were predicted by a target prediction database. miRanda (http://www.microrna.org/microrna/home.do), miRTarBase (http://mirtarbase.mbc.nctu.edu.tw/), and TargetScan (http://www.targetscan.org/) analyses were combined. For a given lncRNA-mRNA pair, both the lncRNA and mRNA were targeted by a common miRNA and negatively coexpressed with this miRNA. Thus, each component of this lncRNA-miRNA-mRNA system was identified as a competing triplet [51].

### 2.10. Quantitative Real-Time PCR (qRT-PCR) Validation

To further improve the ceRNA network reliability, we selected some of the key RNAs in the ceRNA network and used qRT-PCR for validation. RNA samples from MG63 cells treated with different concentrations of baicalin (0 and 50 *μ*g/ml) were collected. Total RNA was extracted using TRIzol Reagent (Invitrogen, CA, USA). cDNA was synthesized from 1.0 *μ*g of total RNA using the PrimeScript RT™ Reagent Kit according to the manufacturer's instructions (TaKaRa, Japan). qRT-PCR was carried out using SYBR Premix Ex Taq™ (TaKaRa) with the Step-One Fast Real-Time PCR system on the CFX Connect™ Real-Time PCR system (Bio-Rad, USA). For quantitative results, the relative expression levels were calculated using the 2^-*ΔΔ*Ct^ method. The PCR conditions were 2 min at 95°C and 40 cycles of 95°C for 5 s and 60°C for 34 s. The primer sequences are shown in Table 1. The data represent the means of three experiments.

### 2.11. Statistical Analysis

In this study, the data are expressed as the mean ± SD. All statistical analyses were performed using SPSS 23.0 (SPSS, Chicago, IL, United States). A *P* value <0.05 was considered to be significant. For sequencing data, we analyzed DEncRNAs and DEmRNAs using Ballgown (Fu J, 2019) software, with *P* < 0.05 and |log2 (fold change) | >2 as screening criteria.

## 3. Results

### 3.1. Baicalin Inhibits Human OS Cell Viability

**Figure 2:**
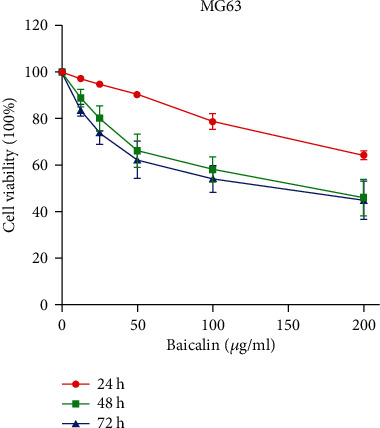
Effect of baicalin treatment on the viability and survival of MG63 cells. MG63 cells were treated with 0, 12.5, 25, 50, 100, and 200 *μ*g/ml baicalin for 24, 48, or 72 h. A cell counting kit-8 (CCK8) assay was used to measure the viability of the treated cells relative to that of the untreated MG63 cells. The data are presented as the mean ± SD of three independent experiments.

To investigate the inhibitory role of baicalin in human OS cell lines (MG63), we employed the CCK-8 assay to determine cell viability. Baicalin was added to the cell medium in a concentration gradient (0, 12.5, 25, 50, 100, and 200 *μ*g/ml) for 24, 48, and 72 h. Baicalin inhibited MG63 viability in a dose- and treatment duration-dependent manner (Figure 2). Our results showed that baicalin significantly inhibits the proliferation of human OS cells.

### 3.2. Baicalin Induces Cell Apoptosis in MG63 Cells

**Figure 3:**
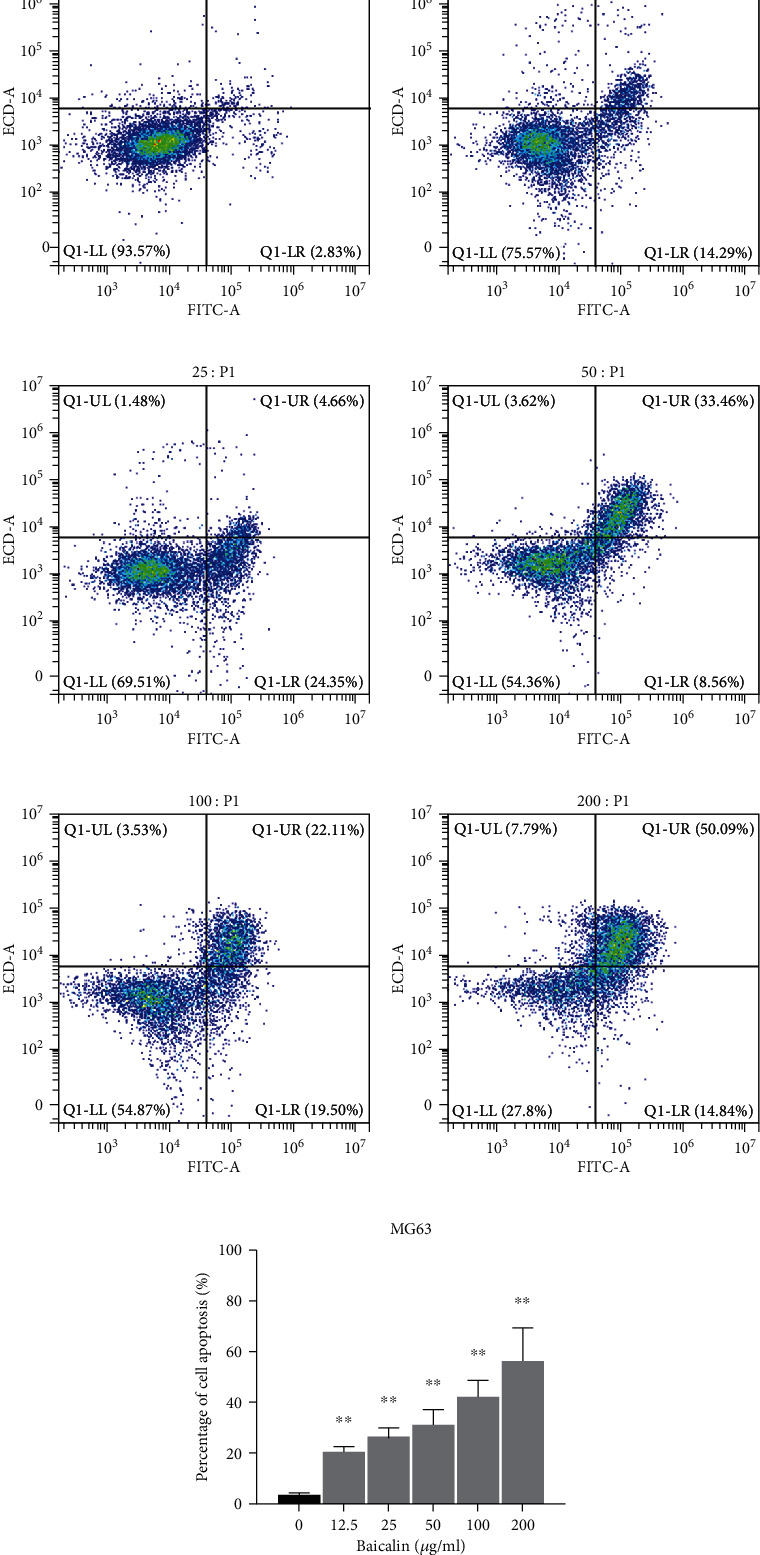
Effect of baicalin treatment on the apoptosis of MG63 cells. Cells were treated with 0, 12.5, 25, 50, 100, and 200 *μ*g/ml baicalin. The percentage of apoptotic cells was measured using Annexin V-PI staining and flow cytometry analysis. The data are presented as the mean ± SD of three independent experiments, ^∗∗^*P* < 0.01.

Cells were treated with 0, 12.5, 25, 50, 100, or 200 *μ*g/ml baicalin and stained with Annexin V-FITC and PI for apoptosis analysis. As shown in Figure 3, the percentage of apoptotic cells was significantly increased in a dose-dependent manner after baicalin treatment. These results demonstrated baicalin-induced cell apoptosis in MG63 cells.

### 3.3. DElncRNAs, DEmiRNAs, and DEmRNAs

**Figure 4:**
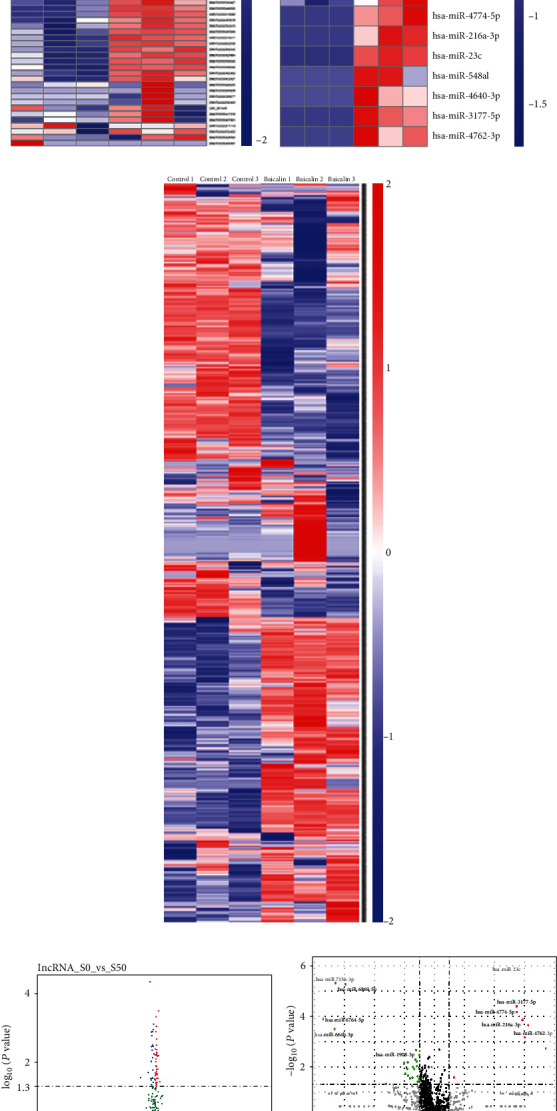
Identification of DElncRNAs, DEmiRNAs, and DEmRNAs in gastric cancer. Hierarchical clustering of DElncRNAs (a), DEmiRNAs (b), and DEmRNAs (c) between untreated and baicalin-untreated MG63 cells. Volcano plot showing DElncRNAs (d), DEmiRNAs (e), and DEmRNAs (f) between untreated and baicalin-treated MG63 cells.

In this study, we screened DElncRNAs, DEmiRNAs, and DEmRNAs using RNA-Seq analysis. Untreated and baicalin-treated MG63 cells were selected for the gene expression assay. The genes with fold changes in the expression > 2.0 and *P* < 0.05 between untreated and baicalin-treated samples were identified as differentially expressed. In total, our project detected 58 lncRNAs, 31 miRNAs, and 2136 mRNAs. Hierarchical clustering and volcano plots show DElncRNAs, DEmiRNAs, and DEmRNAs between untreated and baicalin-treated MG63 cells in Figure 4. There were 58 DElncRNAs (35 upregulated and 23 downregulated), 31 DEmiRNAs (23 upregulated and 8 downregulated), and 2136 DEmRNAs (1023 upregulated and 1113 downregulated) in the baicalin-treated cells compared to the untreated cells.

### 3.4. GO and KEGG Enrichment Analyses of DEmRNAs

**Figure 5:**
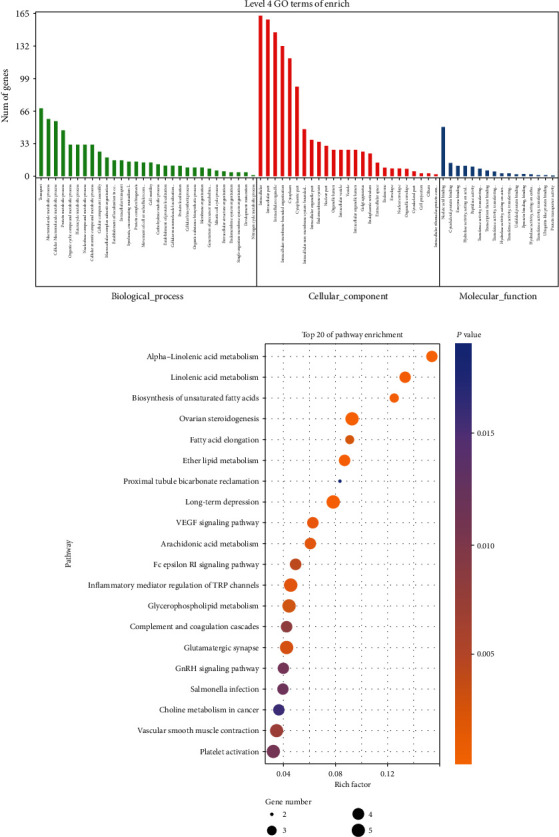
GO and KEGG pathway enrichment analysis of target genes. (a) GO enrichment of mRNAs interacting with lncRNAs. Points of different shapes represent BPs, CCs, and MFs from the GO analysis, and the bar plot shows the number of genes enriched in the GO function. (b) Histogram of KEGG pathway enrichment in baicalin-treated MG63 cells. The size of the dots represents the number of genes annotated in the pathway, and the color of the dots represents the corrected *P* value of the hypergeometric test.

To analyze the biological classification of DEmRNAs, GO and KEGG enrichment analyses were performed. The GO analysis results showed that changes in the biological processes (BPs) of DEGs were mainly enriched in transport, macromolecule metabolic processes, cellular macromolecule metabolic processes, and protein metabolic processes. Changes in the molecular function (MF) were significantly enriched in nucleic acid binding, cytoskeletal protein binding, and enzyme binding. Changes in the cell component (CC) of DEGs were intensively enriched in intracellular and intracellular parts and intracellular organelles (Figure 5(a)). KEGG pathway analysis revealed that the DEmRNAs were mainly enriched in alpha-linolenic acid metabolism, linolenic acid metabolism, biosynthesis of unsaturated fatty acids, and ovarian steroidogenesis (Figure 5(b)).

### 3.5. Construction of a PPI Network from the DEmRNAs

**Figure 6:**
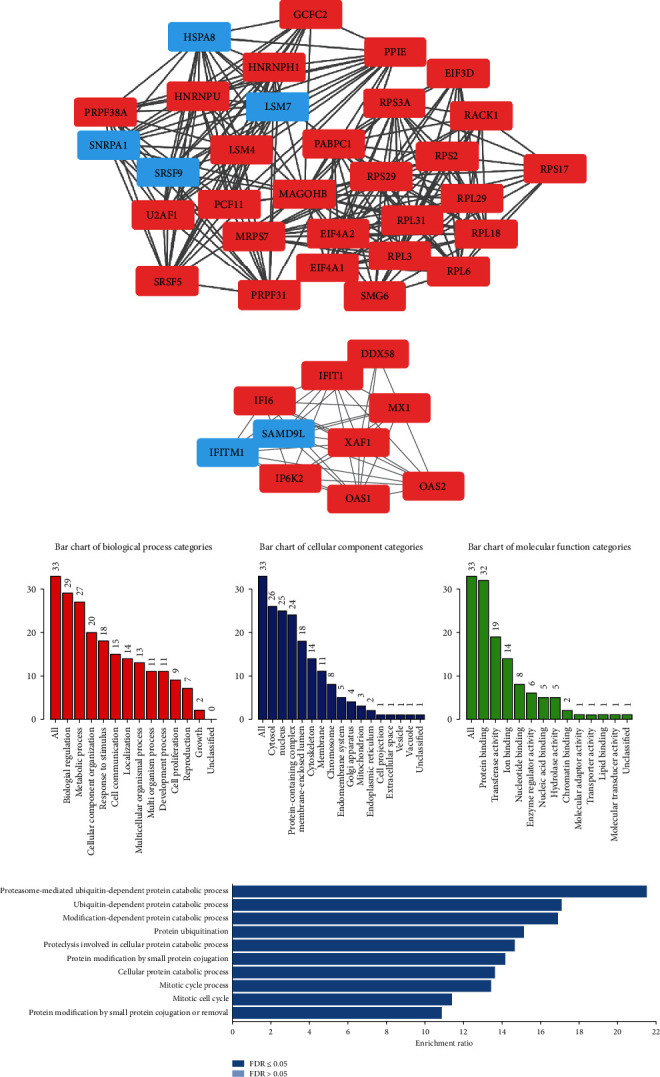
The PPIs of RNA. (a) PPI network. The value of the centrality degree is marked by different node sizes. Up- or downregulation of genes according to the baicalin-untreated MG63 cells vs. control comparison is filled with red or blue, respectively. (b, d) Three hub gene networks were identified by MCODE. (e, f) GO and KEGG pathway enrichment analyses of the hub gene network B. (g, h) GO and KEGG pathway enrichment analyses of the hub gene network C.

The interrelationship between the DEmRNAs was selected from the STRING database to construct the PPI network. A combined score > 0.4 was used as the cutoff criterion. As shown in Figure 6, in total, 148 nodes and 597 edges were included in this PPI network. The nodes denote DEmRNAs, while the edges denote interactions among the DEmRNAs. Two hub gene networks were identified by MCODE. One included CHEK2, TP53BP1, RECQL4, ATM, SMC4, HMMR, CENPE, CCNB1, ECT2, LIG1, FANCI, and OBFC1 (1), and the other included HSPA8, GNB2L1, PRPF38A, RBCK1, PLK1, GFM1, PSMD12, PPIE, RPL6, PABPC1, EIF4A1, ANAPC13, FBXO22, RPL18, BUD31, SNRPA1, PCF11, RPS17, SMC6, and PSMA2 (2). GO enrichment analysis showed that the hub genes might function as G2/M transitions of the mitotic cell cycle (1) and spliceosome complex (2).

### 3.6. Construction of the ceRNA Network

**Figure 7:**
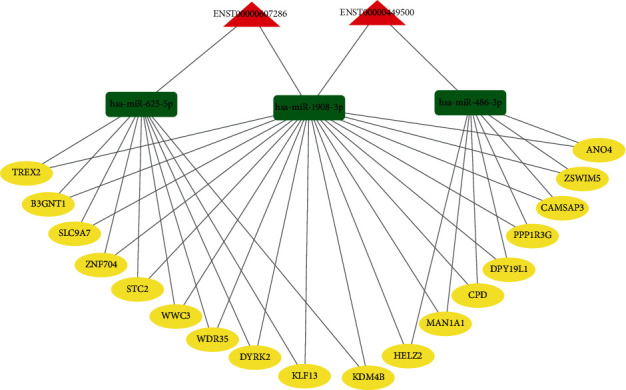
CeRNA network analysis of DElncRNAs, DEmiRNAs, and DEmRNAs in baicalin-treated MG63 cells compared to those in the control group. In the network, red triangles represent lncRNAs, green rectangles represent miRNAs, and yellow circles represent mRNAs.

Many studies have indicated that DElncRNAs could serve as miRNA sponges in organisms. In this study, to better understand the role of lncRNAs in baicalin-treated MG63 cells, we constructed a ceRNA network based on DElncRNA-DEmiRNA-DEmRNA interactions using Cytoscape v 3.6.1 (http://www.cytoscape.org/) software, and the interactions among lncRNAs, miRNAs, and mRNAs were confirmed based on bioinformatic analysis. By using lncRNA as a decoy, miRNA as a center, and mRNA as a target, the lncRNA-miRNA-mRNA regulatory network contained 2 lncRNAs (ENST00000607286 and ENST00000449500), 3 miRNAs (miR-486-3p, miR-1908-3p, and miR-625-5p), and 18 mRNAs (TREX2, B3GNT1, SLC9A7, ZNF704, STC2, WWC3, WDR35, DYRK2, KLF13, KDM4B, HELZ2, MAN1A1, CPD, DPY19L1, PPP1R3G, CAMSAP3, ZSWIM5, and ANO4) (Figure 7).

### 3.7. Expression Profile Validation

**Figure 8:**
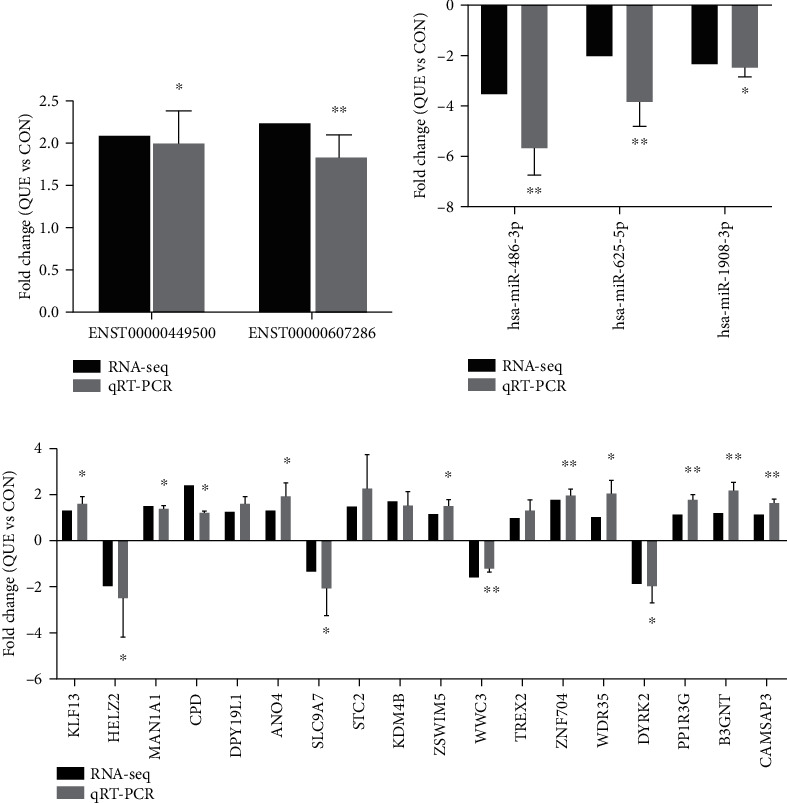
The qRT-PCR results of 2 DElncRNAs (a), 3 DEmiRNAs (b), and 18 DEmRNAs (c) were compared with the sequencing results. The vertical axis represents the mean fold change (FC) (log2 scale) of each RNA measured by qRT-PCR or sequencing. ^∗^*P* < 0.05, ^∗∗^*P* < 0.01, compared with the control (*n* = 3).

To validate the accuracy and reliability of the RNA sequencing results, in total, 23 dysregulated lncRNAs and mRNAs in the ceRNA network were selected for qRT-PCR analysis, including 2 lncRNAs, 3 miRNAs, and 18 mRNAs. As shown in Figure 8, the results from the sequencing data were in agreement with those from qRT-PCR in terms of the expression levels of the validated ncRNAs and mRNAs.

## 4. Discussion

OS is the most common malignant bone disease primarily localized to the long bones and is characterized by a high propensity to metastasize [52]. Baicalin has been widely used to treat various diseases in traditional Chinese medicine, and it has been reported to exert anticancer functions [53]. However, the exact mechanism of its anticancer effects against human OS remains unclear. Moreover, it is vital to identify potential molecular diagnostic markers and/or therapeutic targets to combat human OS.

In recent decades, the complexity of the human genome has been revealed by advanced RNA sequencing analyses, and numerous studies have demonstrated that thousands of lncRNAs are expressed in different kinds of human cancers. Certain lncRNAs behave like oncogenes or tumor suppressors, displaying an important function in cancer initiation, progression, metastasis, and recurrence [54, 55]. To date, only a few lncRNAs have been experimentally verified, but their roles in regulating the gene expression remain to be deciphered. To the best of our knowledge, this is the first study to identify lncRNAs, miRNAs, and mRNAs to reveal regulatory pathways with regard to baicalin-induced apoptosis in MG63 cells.

As shown by the results, baicalin inhibited the proliferation and induced the apoptosis of MG63 cells. With |log2 (fold change) | >2 and *P* value <0.05 thresholds, a total of 58 lncRNAs, 31 miRNAs, and 2136 mRNAs with significant differential expression were identified in the baicalin-treated MG63 cells compared with the untreated cells. A deeper understanding of the antitumor effects in OS was provided in this study. Bioinformatic analysis was performed to explore interactions among the DEmRNAs. The most enriched GO terms of DEmRNAs contained BPs, MFs, and CCs, which included transport, cellular nitrogen compound metabolism, macromolecule metabolic processes, intracellular parts, and intracellular and nucleic acid binding. These GO terms could participate in DNA replication, damage detection, and regulation of the activity of cyclin-dependent protein serine/threonine kinases. Through KEGG pathway analysis, we detected a number of cancer-related pathways, including alpha-linolenic acid metabolism, linoleic acid metabolism, biosynthesis of unsaturated fatty acids, the VEGF signaling pathway, and choline metabolism in cancer. Studies have shown that targeting the VEGF-VEGFR pathway seems to be the best approach in hepatic epithelioid hemangioendothelioma [56].

Abnormal choline metabolism continues to be identified in multiple cancers [57]. The above views demonstrate that our ceRNA network reflects vital mechanisms of anticancer effects. Furthermore, we then constructed a PPI network to identify hub DEmRNAs. Proteins that corresponded to the genes were used to build the PPI network. A PPI network including 148 proteins and 597 edges was constructed to reveal the relationships among baicalin-treated proteins in MG63 cells. Two core networks containing a total of 64 hub genes were identified in this PPI network by using MCODE v1.5.1. GO enrichment analysis showed that the hub genes might function in proteasome-mediated ubiquitin-dependent protein catabolic processes and nuclear-transcribed mRNA catabolic processes, indicating that these processes are present in OS. These reports, together with our findings, suggest that these key regulators might play key roles in regulating baicalin-induced apoptosis in human OS cells.

In addition, to provide a possible explanation for the baicalin-induced altered RNA expression levels, we established a ceRNA network of lncRNA-miRNA-mRNA according to bioinformatic analysis. The crosstalk between 2 lncRNAs, 3 miRNAs, and 18 mRNAs revealed a complex mechanism in baicalin-treated MG63 cells. The ceRNA hypothesis explains a new mechanism of RNA interaction and provides important clues and theoretical guidance for further understanding the tumorigenesis mechanism [58]. We observed that lncRNAs sponge several miRNAs, while miRNAs could regulate more than one mRNA. Based on the ceRNA network, we concluded that the lncRNAs with changed expression were linked with baicalin-treated MG63 cells by sponging the related miRNAs. Such a sponging effect may reflect the regulatory potential of ncRNAs in MG63 cells. In recent years, increasing evidence has indicated that ceRNAs are related to the development of cancers [59–61].

LncRNAs, which comprise the widest ncRNA subgroup, are RNA molecules of more than 200 bases in length, transcribed by RNA polymerase II, and capped and polyadenylated at their 5′ and 3′ ends, respectively [62]. LncRNAs exert both beneficial and detrimental functions by acting at the transcriptional, posttranscriptional, or epigenetic level. In our study, we also identified two DElncRNAs (ENST00000607286 and ENST00000449500) in the ceRNA networks, which have rarely been studied in previously. ENST00000607286 is a transcript of two exons with a length of 2204 nucleotides and is located on chromosome 2q13. Our results showed interactions between ENST00000607286 and miR-625-5p and miR-1908-3p. Studies have shown that the aberrant expression of miR-625-5p in cancers might be a potential risk factor. The mechanisms underlying the antitumor function of miR-625-5p in different cancer types have been demonstrated in previous studies. LINC00958 facilitates cervical cancer cell proliferation and metastasis by sponging miR-625-5p to upregulate the LRRC8E expression, which provides a novel biomarker for experimenters to discover better treatments for cervical cancer patients [63]. LINC009581 elevated the expression of CPSF7 by acting as a miR-625-5p sponge, which accelerated the development and progression of lung adenocarcinoma, thereby demonstrating that LINC009581 might be utilized as a promising therapeutic target for lung adenocarcinoma [64]. LINC00511 is a tumor promoter that sponges miR-625-5p by targeting NFIX in gastric cancer cells and could be considered a brand new target for gastric cancer treatment [65]. Novel hypoxia-associated circDENND2A enhances the migration and invasion of glioma cells by directly sponging miR-625-5p [66].

In addition, ENST00000449500 is located on chromosome 20, with a total length of 801 bp and two exons. Our results also showed intimate interactions between ENST00000449500 and miRNAs, such as miR-486-3p and miR-1908-3p. Unlike lncRNAs, miRNAs, which are a group of endogenous, evolutionarily conserved nonprotein-coding RNA molecules with typical lengths of 20-24 nucleotides, essentially regulate the gene expression via posttranscriptional regulation. ceRNAs can competitively bind to MREs, revealing that miRNAs are at the center of ceRNA networks. It has been reported that dysregulated miRNAs play various roles in the initiation, progression, invasiveness, and metastasis of tumors [67]. miRNAs are involved in multiple roles during carcinogenesis. Among them, miR-486-3p has been shown to exert a regulatory role in tumor progression. Chou et al. [68] demonstrated that miRNA-486-3p functions as a tumor suppressor in oral cancer by targeting DDR1. Another study on cervical cancer suggested that c-Myc could upregulate the lncRNA-PVT1 expression, which subsequently releases the inhibition of ECM1 by sponging miR-486-3p, thus enhancing the proliferation and viability of cervical cancer cells. The c-Myc/lncRNA-PVT1/miR-486-3p/ECM1 axis might serve as a new target for more efficient diagnosis and treatment of cervical cancer [69]. In laryngeal squamous cell carcinoma (LSCC), circFLNA functions in LSCC migration by sponging miR-486-3p which downregulate the FLNA protein expression. Targeting the circFLNA/miR-486-3p/FLAN axis provides a potential therapeutic target for aggressive LSCC [70]. Moreover, miR-486-3p may serve as a biomarker for the detection of oral tongue squamous cell carcinoma [71]. To date, no study has reported any association of miR-1908-3p with cancer. Moreover, our studies have reported that the apoptosis-related miRNAs miR-130a, miR-222, miR-195, miR-29c, miR-92a-1, and miR-216a were aberrantly expressed in baicalin-induced apoptosis in MG63 cells. Interestingly, among them, miR-195, miR-29c, and miR-92a-1 were reported in a previous study of osteosarcoma [72–74], indicating the high degree of confidence in our sequencing results. This is the first study to show the aberrant expression of ENST00000449500 and ENST00000607286 in MG63 cells and indicates a potential prognostic role of this 2-lncRNA signature in MG63 cells. In addition, bioinformatic-based investigations of lncRNAs will be helpful in future experimental studies.

Eventually, the selected RNAs in the ceRNA network were verified by qRT-PCR to confirm the reliability and validity of the above bioinformatic results. The 18 verified RNA expression levels were in line with the sequencing results, indicating a high degree of confidence of this network. We verified the independent RNAs, and the results supported our findings and provided a better understanding of lncRNA-related ceRNAs and their important role in baicalin-induced apoptosis of MG63 cells.

ENST00000449500 is also called lncRNA melanoma highly expressed noncoding RNA (MHENCR). Chen et al. [75] found that MHENCR promotes melanoma progression by regulating the miR-425/489-mediated PI3K-Akt pathway. According to our research, lncRNA MHENCR might affect the OS process by regulating miR-1908-3p/miR-468-3p, indicating a new mechanism of MHENCR in OS. Another lncRNA, ENST00000607286, named SLC9A3-AS1 on NCBI, was one of thirteen transcripts. Bai et al. [76] found that SLC9A3-AS1 was highly expressed in the peripheral blood of lung cancer patients, but there is no research on its function thus far.

To our knowledge, our work is the first expression profile to analyze and validate the ceRNA-mediated mechanism to signify how baicalin exerts its antitumor effects in OS. Although the findings of our study have important clinical implications, the limitations must also be noted. First, a comparative analysis of the two groups of ceRNA networks showed only two DElncRNAs and three DEmiRNAs. Second, ceRNA binding experiments also need to be further investigated. Further experiments are needed to support the identification of functional roles.

## 5. Conclusions

In summary, the present study demonstrated that baicalin can reduce cell viability and induce cell apoptosis in MG63 cells. DEncRNAs, DEmiRNAs, and DEmRNAs were identified, and a functional lncRNA-miRNA-mRNA ceRNA regulatory network for MG63 cells without and with baicalin intervention was successfully constructed. Bioinformatic analysis may provide a better understanding of the potential roles of RNAs in human OS cells treated with baicalin, and these RNAs might serve as prognostic biomarkers and therapeutic targets. Two DElncRNAs (ENST00000607286 and ENST00000449500), 3 DEmiRNAs (miR-486-3p, miR-1908-3p, and miR-625-5p) and 18 DEmRNAs (TREX2, B3GNT1, SLC9A7, ZNF704, STC2, WWC3, WDR35, DYRK2, KLF13, KDM4B, HELZ2, MAN1A1, CPD, DPY19L1, PPP1R3G, CAMSAP3, ZSWIM5, and ANO4) were identified in the ceRNA network as closely associated with OS pathogenesis. The ceRNA regulatory network might illuminate the inner molecular mechanism involved in the tumorigenesis and progression of OS. Moreover, the corresponding roles and molecular mechanisms of these ncRNAs and mRNAs need to be further elucidated.

## Figures and Tables

**Table 1:** Primer sequences used for qRT-PCR analysis.

Gene name		Primer sequence (5′-3′)
*lncRNA*		
ENST00000607286	Forward	GGTTCAGCTCGTGGAAGACA
	Reverse	TTTAAACGCGCCTACAGGGT

ENST00000449500	Forward	GATGGAACCCTGACCCTGTG
	Reverse	GACACACATAGACCGCGTGA

GAPDH	Forward	CAGCCTCAAGATCATCAGCA
	Reverse	ACAGTCTTCTGGGTGGCAGT

*miRNA*		
miR-486-3p	Forward	AACAAGTCGGGGCAGCTCA
	Reverse	GTCGTATCCAGTGCAGGGTCC
	RT	GTCGTATCCAGTGCAGGGTCCGAGGTATTCGCACTGGATACGACATCCTGT

miR-1908-3p	Forward	ACCGGCCGCCGGCTCC
	Reverse	AGTGCAGGGTCCGAGGTATT
	RT	GTCGTATCCAGTGCAGGGTCCGAGGTATTCGCACTGGATACGACCGGGGC

miR-625-5p	Forward	AACCGGAGGGGGAAAGTTC
	Reverse	GTCGTATCCAGTGCAGGGT
	RT	GTCGTATCCAGTGCAGGGTCCGAGGTATTCGCACTGGATACGACGGACTA

U6	Forward	CTCGCTTCGGCAGCACA
	Reverse	AACGCTTCACGAATTTGCGT

*mRNA*		
TREX2	Forward	CACCTGATCTCCAGTGACGG
	Reverse	TGGGGCCAGTTACACAAAGG

B3GNT1	Forward	TGCTCCCGGACAAGATATGAG
	Reverse	TGCCATCATCAGGATACCCA

SLC9A7	Forward	TGACTGGTGTTGTGACTGCT
	Reverse	AACGTGCTCCAGGACATGAG

ZNF704	Forward	TCTTCAGCAAAGCTCCCTGG
	Reverse	AACGTGCTCCAGGACATGAG

STC2	Forward	TGTAGTAGTTGAGCGCAGGC
	Reverse	AAGGAGTCGAGCAGGTGTTG

WWC3	Forward	CCCCGAGAAATTTCAGCCCT
	Reverse	CGTGCCACTCCGAACAAAAG

WDR35	Forward	ATGGAGACATTTGGTGCAACG
	Reverse	GAGGCTGCTATCACATGGGT

DYRK2	Forward	GTTCGTCAGCTTCAGGCTTC
	Reverse	CTTACTGCCGCCAATCGTGT

KLF13	Forward	ACGGGCGAGAAGAAGTTCAG
	Reverse	GCATTCCCGGGTGGAAGTTG

KDM4B	Forward	ACCATCACTGTTGCTGGAGG
	Reverse	CACTTCTGGATGGCGAGGTT
HELZ2	Forward	CATCGCAGGTCCCCATCTAC
	Reverse	ATTTGGACCCAGAAGAGCCG

MAN1A1	Forward	AGCCCAGCCTAGGAAAGAGG
	Reverse	GGGAGACTCGTCAACTTCGC

CPD	Forward	AGATTGTCTAAAGCATGGCAGT
	Reverse	TTCACACTTCCTGTAGCAGTT

DPY19L1	Forward	CTGGACCACGCTCCTGTTAG
	Reverse	TGCGAAAAGCCATCTCCCTT

PPP1R3G	Forward	ATCATTGTGTCAGGCAGGGG
	Reverse	AAGCCAATTCAAACGGGTGC

CAMSAP3	Forward	CTTTTCTGGGTGGACACGAC
	Reverse	TTGCGGTATCGGATCGAGG

ZSWIM5	Forward	TTCCCCAGAGTGCCATTCAC
	Reverse	GCCAATGTAATTCACGCCCC

ANO4	Forward	ATCACTTTGCTGGCCTCCTC
	Reverse	TTCCTCAGTGCCTTGGTGTC

## Data Availability

The datasets used and/or analyzed during the current study are available from the corresponding author on reasonable request.

## Abbreviations

BA: Baicalin
OS: Osteosarcoma
ceRNA: Competitive endogenous RNA network.
